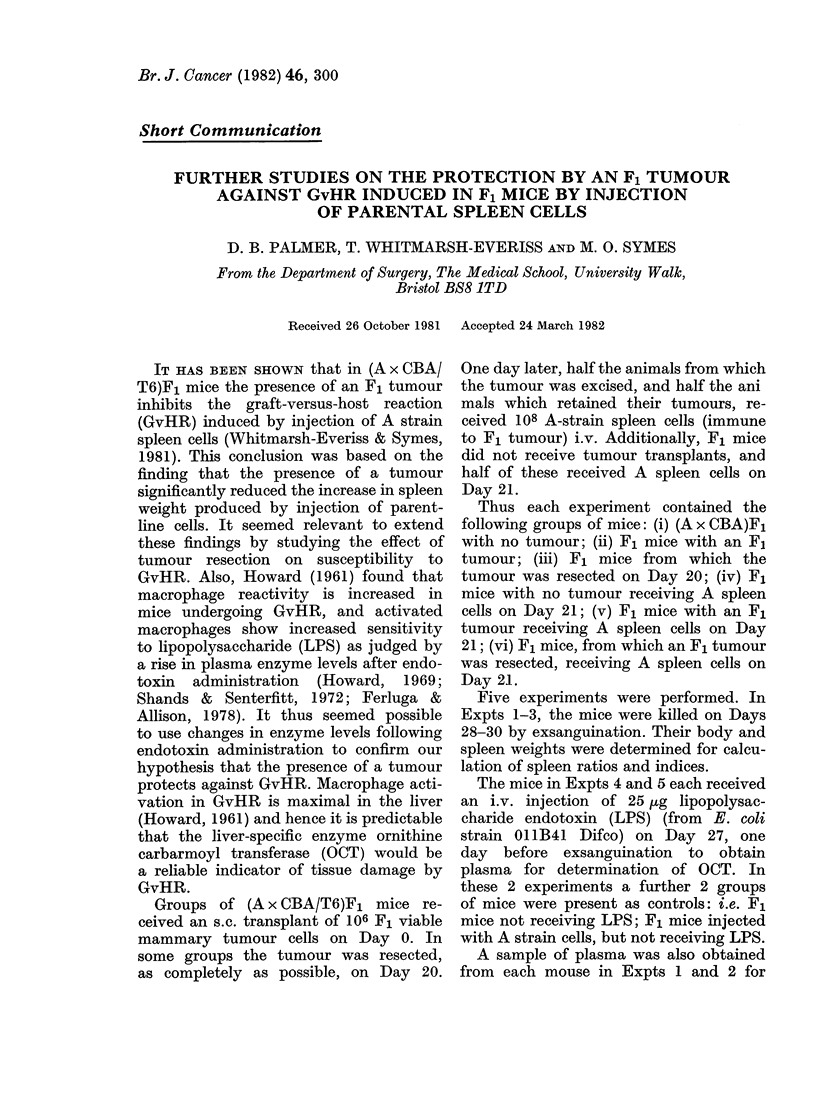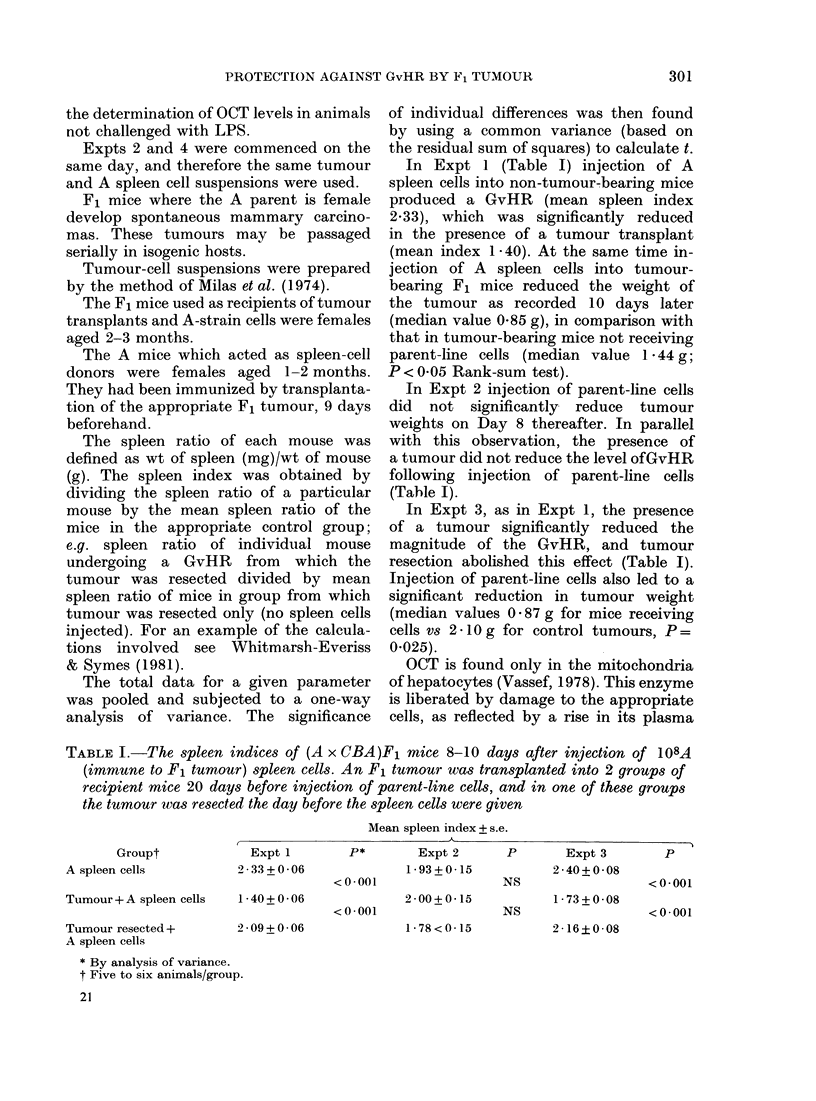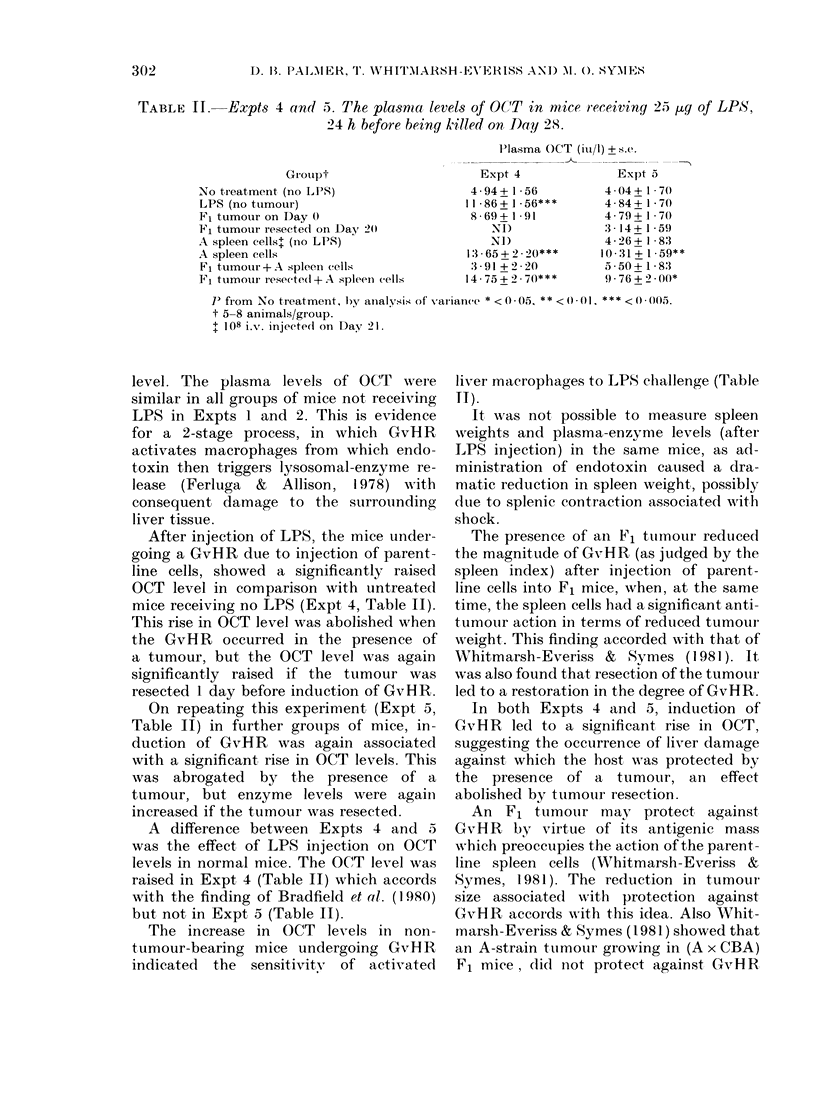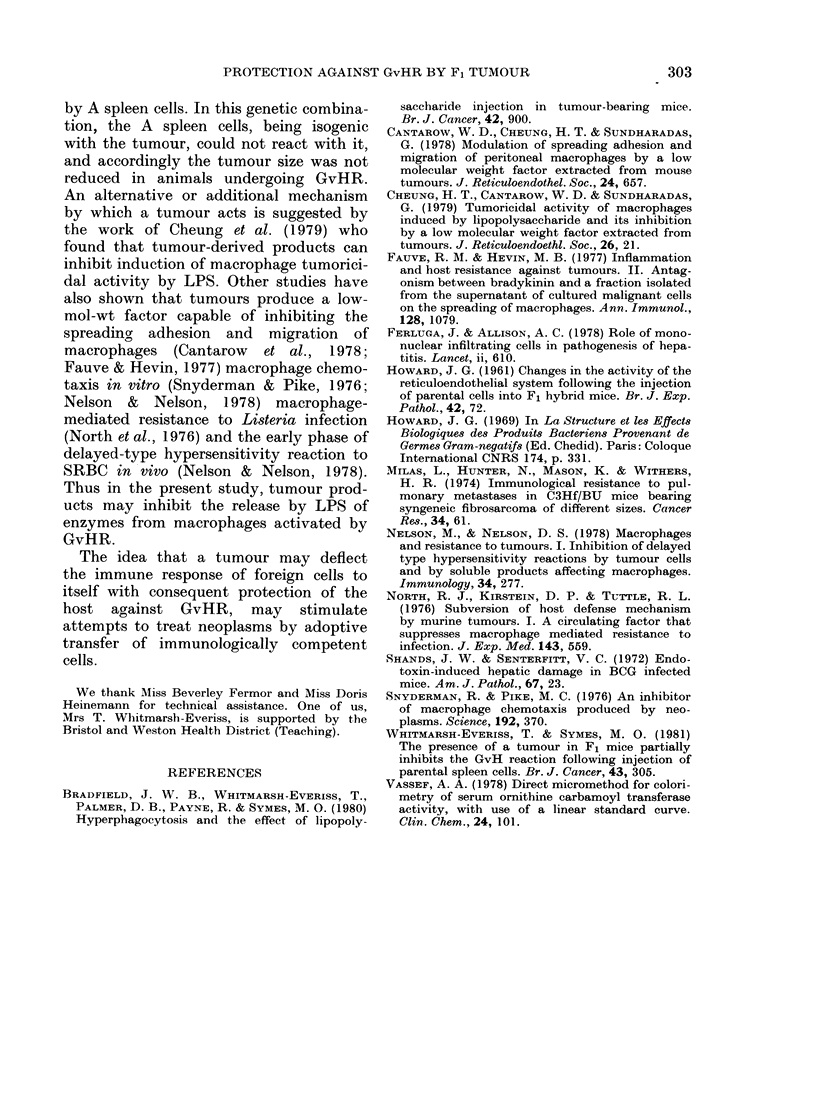# Further studies on the protection by an F1 tumour against GvHR induced in F1 mice by injection of parental spleen cells.

**DOI:** 10.1038/bjc.1982.198

**Published:** 1982-08

**Authors:** D. B. Palmer, T. Whitmarsh-Everiss, M. O. Symes


*Br. J. Cancer* (1982) **46**, 300

***Short Communication***

### FURTHER STUDIES ON THE PROTECTION BY AN $F_1$ TUMOUR AGAINST GvHR INDUCED IN $F_1$ MICE BY INJECTION OF PARENTAL SPLEEN CELLS

D. B. PALMER, T. WHITMARSH-EVERISS AND M. O. SYMES

*From the Department of Surgery, The Medical School, University Walk, Bristol BS8 1TD*

Received 26 October 1981 Accepted 24 March 1982

IT HAS BEEN SHOWN that in (A × CBA/T6)$F_1$ mice the presence of an $F_1$ tumour inhibits the graft-versus-host reaction (GvHR) induced by injection of A strain spleen cells (Whitmarsh-Everiss & Symes, 1981). This conclusion was based on the finding that the presence of a tumour significantly reduced the increase in spleen weight produced by injection of parent-line cells. It seemed relevant to extend these findings by studying the effect of tumour resection on susceptibility to GvHR. Also, Howard (1961) found that macrophage reactivity is increased in mice undergoing GvHR, and activated macrophages show increased sensitivity to lipopolysaccharide (LPS) as judged by a rise in plasma enzyme levels after endotoxin administration (Howard, 1969; Shands & Senterfitt, 1972; Ferluga & Allison, 1978). It thus seemed possible to use changes in enzyme levels following endotoxin administration to confirm our hypothesis that the presence of a tumour protects against GvHR. Macrophage activation in GvHR is maximal in the liver (Howard, 1961) and hence it is predictable that the liver-specific enzyme ornithine carbarmoyl transferase (OCT) would be a reliable indicator of tissue damage by GvHR.

Groups of (A × CBA/T6)$F_1$ mice received an s.c. transplant of $10^6$ $F_1$ viable mammary tumour cells on Day 0. In some groups the tumour was resected, as completely as possible, on Day 20. One day later, half the animals from which the tumour was excised, and half the animals which retained their tumours, received $10^8$ A-strain spleen cells (immune to $F_1$ tumour) i.v. Additionally, $F_1$ mice did not receive tumour transplants, and half of these received A spleen cells on Day 21.

Thus each experiment contained the following groups of mice: (i) (A × CBA)$F_1$ with no tumour; (ii) $F_1$ mice with an $F_1$ tumour; (iii) $F_1$ mice from which the tumour was resected on Day 20; (iv) $F_1$ mice with no tumour receiving A spleen cells on Day 21; (v) $F_1$ mice with an $F_1$ tumour receiving A spleen cells on Day 21; (vi) $F_1$ mice, from which an $F_1$ tumour was resected, receiving A spleen cells on Day 21.

Five experiments were performed. In Expts 1–3, the mice were killed on Days 28–30 by exsanguination. Their body and spleen weights were determined for calculation of spleen ratios and indices.

The mice in Expts 4 and 5 each received an i.v. injection of 25 $\mu$g lipopolysaccharide endotoxin (LPS) (from *E. coli* strain 011B41 Difco) on Day 27, one day before exsanguination to obtain plasma for determination of OCT. In these 2 experiments a further 2 groups of mice were present as controls: *i.e.* $F_1$ mice not receiving LPS; $F_1$ mice injected with A strain cells, but not receiving LPS.

A sample of plasma was also obtained from each mouse in Expts 1 and 2 for the determination of OCT levels in animals not challenged with LPS.

Expts 2 and 4 were commenced on the same day, and therefore the same tumour and A spleen cell suspensions were used.

$F_1$ mice where the A parent is female develop spontaneous mammary carcinomas. These tumours may be passaged serially in isogenic hosts.

Tumour-cell suspensions were prepared by the method of Milas *et al.* (1974).

The $F_1$ mice used as recipients of tumour transplants and A-strain cells were females aged 2–3 months.

The A mice which acted as spleen-cell donors were females aged 1–2 months. They had been immunized by transplantation of the appropriate $F_1$ tumour, 9 days beforehand.

The spleen ratio of each mouse was defined as wt of spleen (mg)/wt of mouse (g). The spleen index was obtained by dividing the spleen ratio of a particular mouse by the mean spleen ratio of the mice in the appropriate control group; *e.g.* spleen ratio of individual mouse undergoing a GvHR from which the tumour was resected divided by mean spleen ratio of mice in group from which tumour was resected only (no spleen cells injected). For an example of the calculations involved see Whitmarsh-Everiss & Symes (1981).

The total data for a given parameter was pooled and subjected to a one-way analysis of variance. The significance of individual differences was then found by using a common variance (based on the residual sum of squares) to calculate $t$.

In Expt 1 (Table I) injection of A spleen cells into non-tumour-bearing mice produced a GvHR (mean spleen index 2·33), which was significantly reduced in the presence of a tumour transplant (mean index 1·40). At the same time injection of A spleen cells into tumour-bearing $F_1$ mice reduced the weight of the tumour as recorded 10 days later (median value 0·85 g), in comparison with that in tumour-bearing mice not receiving parent-line cells (median value 1·44 g; $P < 0{\cdot}05$ Rank-sum test).

In Expt 2 injection of parent-line cells did not significantly reduce tumour weights on Day 8 thereafter. In parallel with this observation, the presence of a tumour did not reduce the level of GvHR following injection of parent-line cells (Table I).

In Expt 3, as in Expt 1, the presence of a tumour significantly reduced the magnitude of the GvHR, and tumour resection abolished this effect (Table I). Injection of parent-line cells also led to a significant reduction in tumour weight (median values 0·87 g for mice receiving cells *vs* 2·10 g for control tumours, $P =$ 0·025).

OCT is found only in the mitochondria of hepatocytes (Vassef, 1978). This enzyme is liberated by damage to the appropriate cells, as reflected by a rise in its plasma

TABLE I.—*The spleen indices of* $(A \times CBA)F_1$ *mice* 8–10 *days after injection of* $10^8 A$ *(immune to* $F_1$ *tumour) spleen cells. An* $F_1$ *tumour was transplanted into* 2 *groups of recipient mice* 20 *days before injection of parent-line cells, and in one of these groups the tumour was resected the day before the spleen cells were given*

| | Mean spleen index ± s.e. | | | | | |
|---|---|---|---|---|---|---|
| Group† | Expt 1 | P* | Expt 2 | P | Expt 3 | P |
| A spleen cells | 2·33 ± 0·06 | | 1·93 ± 0·15 | | 2·40 ± 0·08 | |
| | | < 0·001 | | NS | | < 0·001 |
| Tumour + A spleen cells | 1·40 ± 0·06 | | 2·00 ± 0·15 | | 1·73 ± 0·08 | |
| | | < 0·001 | | NS | | < 0·001 |
| Tumour resected + A spleen cells | 2·09 ± 0·06 | | 1·78 < 0·15 | | 2·16 ± 0·08 | |

* By analysis of variance.

† Five to six animals/group.

TABLE II.—*Expts 4 and 5. The plasma levels of OCT in mice receiving 25 μg of LPS, 24 h before being killed on Day 28.*

| Group† | Plasma OCT (iu/l) ± s.e. Expt 4 | Expt 5 |
|---|---|---|
| No treatment (no LPS) | 4·94 ± 1·56 | 4·04 ± 1·70 |
| LPS (no tumour) | 11·86 ± 1·56*** | 4·84 ± 1·70 |
| $F_1$ tumour on Day 0 | 8·69 ± 1·91 | 4·79 ± 1·70 |
| $F_1$ tumour resected on Day 20 | ND | 3·14 ± 1·59 |
| A spleen cells‡ (no LPS) | ND | 4·26 ± 1·83 |
| A spleen cells | 13·65 ± 2·20*** | 10·31 ± 1·59** |
| $F_1$ tumour + A spleen cells | 3·91 ± 2·20 | 5·50 ± 1·83 |
| $F_1$ tumour resected + A spleen cells | 14·75 ± 2·70*** | 9·76 ± 2·00* |

*P* from No treatment, by analysis of variance * < 0·05, ** < 0·01, *** < 0·005.

† 5–8 animals/group.

‡ $10^8$ i.v. injected on Day 21.

level. The plasma levels of OCT were similar in all groups of mice not receiving LPS in Expts 1 and 2. This is evidence for a 2-stage process, in which GvHR activates macrophages from which endotoxin then triggers lysosomal-enzyme release (Ferluga & Allison, 1978) with consequent damage to the surrounding liver tissue.

After injection of LPS, the mice undergoing a GvHR due to injection of parent-line cells, showed a significantly raised OCT level in comparison with untreated mice receiving no LPS (Expt 4, Table II). This rise in OCT level was abolished when the GvHR occurred in the presence of a tumour, but the OCT level was again significantly raised if the tumour was resected 1 day before induction of GvHR.

On repeating this experiment (Expt 5, Table II) in further groups of mice, induction of GvHR was again associated with a significant rise in OCT levels. This was abrogated by the presence of a tumour, but enzyme levels were again increased if the tumour was resected.

A difference between Expts 4 and 5 was the effect of LPS injection on OCT levels in normal mice. The OCT level was raised in Expt 4 (Table II) which accords with the finding of Bradfield *et al.* (1980) but not in Expt 5 (Table II).

The increase in OCT levels in non-tumour-bearing mice undergoing GvHR indicated the sensitivity of activated liver macrophages to LPS challenge (Table II).

It was not possible to measure spleen weights and plasma-enzyme levels (after LPS injection) in the same mice, as administration of endotoxin caused a dramatic reduction in spleen weight, possibly due to splenic contraction associated with shock.

The presence of an $F_1$ tumour reduced the magnitude of GvHR (as judged by the spleen index) after injection of parent-line cells into $F_1$ mice, when, at the same time, the spleen cells had a significant anti-tumour action in terms of reduced tumour weight. This finding accorded with that of Whitmarsh-Everiss & Symes (1981). It was also found that resection of the tumour led to a restoration in the degree of GvHR.

In both Expts 4 and 5, induction of GvHR led to a significant rise in OCT, suggesting the occurrence of liver damage against which the host was protected by the presence of a tumour, an effect abolished by tumour resection.

An $F_1$ tumour may protect against GvHR by virtue of its antigenic mass which preoccupies the action of the parent-line spleen cells (Whitmarsh-Everiss & Symes, 1981). The reduction in tumour size associated with protection against GvHR accords with this idea. Also Whitmarsh-Everiss & Symes (1981) showed that an A-strain tumour growing in (A × CBA) $F_1$ mice, did not protect against GvHR

by A spleen cells. In this genetic combination, the A spleen cells, being isogenic with the tumour, could not react with it, and accordingly the tumour size was not reduced in animals undergoing GvHR. An alternative or additional mechanism by which a tumour acts is suggested by the work of Cheung *et al.* (1979) who found that tumour-derived products can inhibit induction of macrophage tumoricidal activity by LPS. Other studies have also shown that tumours produce a low-mol-wt factor capable of inhibiting the spreading adhesion and migration of macrophages (Cantarow *et al.*, 1978; Fauve & Hevin, 1977) macrophage chemotaxis *in vitro* (Snyderman & Pike, 1976; Nelson & Nelson, 1978) macrophage-mediated resistance to *Listeria* infection (North *et al.*, 1976) and the early phase of delayed-type hypersensitivity reaction to SRBC *in vivo* (Nelson & Nelson, 1978). Thus in the present study, tumour products may inhibit the release by LPS of enzymes from macrophages activated by GvHR.

The idea that a tumour may deflect the immune response of foreign cells to itself with consequent protection of the host against GvHR, may stimulate attempts to treat neoplasms by adoptive transfer of immunologically competent cells.

We thank Miss Beverley Fermor and Miss Doris Heinemann for technical assistance. One of us, Mrs T. Whitmarsh-Everiss, is supported by the Bristol and Weston Health District (Teaching).

### REFERENCES

BRADFIELD, J. W. B., WHITMARSH-EVERISS, T., PALMER, D. B., PAYNE, R. & SYMES, M. O. (1980) Hyperphagocytosis and the effect of lipopolysaccharide injection in tumour-bearing mice. *Br. J. Cancer*, **42**, 900.

CANTAROW, W. D., CHEUNG, H. T. & SUNDHARADAS, G. (1978) Modulation of spreading adhesion and migration of peritoneal macrophages by a low molecular weight factor extracted from mouse tumours. *J. Reticuloendothel. Soc.*, **24**, 657.

CHEUNG, H. T., CANTAROW, W. D. & SUNDHARADAS, G. (1979) Tumoricidal activity of macrophages induced by lipopolysaccharide and its inhibition by a low molecular weight factor extracted from tumours. *J. Reticuloendoethl. Soc.*, **26**, 21.

FAUVE, R. M. & HEVIN, M. B. (1977) Inflammation and host resistance against tumours. II. Antagonism between bradykinin and a fraction isolated from the supernatant of cultured malignant cells on the spreading of macrophages. *Ann. Immunol.*, **128**, 1079.

FERLUGA, J. & ALLISON, A. C. (1978) Role of mononuclear infiltrating cells in pathogenesis of hepatitis. *Lancet*, ii, 610.

HOWARD, J. G. (1961) Changes in the activity of the reticuloendothelial system following the injection of parental cells into $F_1$ hybrid mice. *Br. J. Exp. Pathol.*, **42**, 72.

HOWARD, J. G. (1969) In *La Structure et les Effects Biologiques des Produits Bacteriens Provenant de Germes Gram-negatifs* (Ed. Chedid). Paris: Coloque International CNRS 174, p. 331.

MILAS, L., HUNTER, N., MASON, K. & WITHERS, H. R. (1974) Immunological resistance to pulmonary metastases in C3Hf/BU mice bearing syngeneic fibrosarcoma of different sizes. *Cancer Res.*, **34**, 61.

NELSON, M., & NELSON, D. S. (1978) Macrophages and resistance to tumours. I. Inhibition of delayed type hypersensitivity reactions by tumour cells and by soluble products affecting macrophages. *Immunology*, **34**, 277.

NORTH, R. J., KIRSTEIN, D. P. & TUTTLE, R. L. (1976) Subversion of host defense mechanism by murine tumours. I. A circulating factor that suppresses macrophage mediated resistance to infection. *J. Exp. Med.* **143**, 559.

SHANDS, J. W. & SENTERFITT, V. C. (1972) Endotoxin-induced hepatic damage in BCG infected mice. *Am. J. Pathol.*, **67**, 23.

SNYDERMAN, R. & PIKE, M. C. (1976) An inhibitor of macrophage chemotaxis produced by neoplasms. *Science*, **192**, 370.

WHITMARSH-EVERISS, T. & SYMES, M. O. (1981) The presence of a tumour in $F_1$ mice partially inhibits the GvH reaction following injection of parental spleen cells. *Br. J. Cancer*, **43**, 305.

VASSEF, A. A. (1978) Direct micromethod for colorimetry of serum ornithine carbamoyl transferase activity, with use of a linear standard curve. *Clin. Chem.*, **24**, 101.